# Repeatability of Ocular Measurements with a Dual-Scheimpflug Analyzer in Healthy Eyes

**DOI:** 10.1155/2014/808646

**Published:** 2014-04-24

**Authors:** Carmen Lopez de la Fuente, Ana Sanchez-Cano, Francisco Segura, Lorena Fuentes-Broto, Isabel Pinilla

**Affiliations:** ^1^Department of Applied Physics, University of Zaragoza, C/Pedro Cerbuna 12, 50009 Zaragoza, Spain; ^2^Aragon Health Sciences Institute, Avenida San Juan Bosco, 13, 50009 Zaragoza, Spain; ^3^Department of Surgery, Gynecology and Obstetrics, University of Zaragoza, C/Pedro Cerbuna 12, 50009 Zaragoza, Spain; ^4^Department of Physiology and Pharmacology, University of Zaragoza, C/Pedro Cerbuna 12, 50009 Zaragoza, Spain; ^5^Department of Ophthalmology, Lozano Blesa University Hospital, Avenida San Juan Bosco, 15, 50009 Zaragoza, Spain

## Abstract

*Purpose*. To assess the repeatability of the Galilei dual Scheimpflug analyzer (GDSA) in anterior segment examination. *Methods*. Fifty-two eyes from 52 healthy volunteers were prospectively and consecutively recruited. Anatomic, axial, refractive, and instantaneous parameters were measured with GDSA to provide a complete characterization of the anterior segment. Repeatability was assessed calculating intraclass correlation coefficient (ICC), and coefficient of variation (COV). *Results*. Correlation among repeated measurements showed almost perfect reliability (ICC > 0.81) for all parameters except thinnest central corneal thickness (CCT) (0.78), corneal thickness average out (0.79), and posterior axial curvature average out (0.60). Repeatability was excellent (COV < 10%) for all parameters except anterior chamber volume and, superior iridocorneal angle and eccentricities. In these last three parameters, repeatability limits were excessively high compared to the mean. *Conclusions*. GDSA in healthy young persons had an almost perfect correlation in measuring anatomic, axial, instantaneous, and refractive parameters with greater variability for peripheral terms. Repeatability of anatomical parameters like pachymetry, anterior chamber, or iridocorneal angle and eccentricity were limited. In healthy young persons, the other evaluated parameters had very good repeatability and their limits of agreement showed excellent clinical results for this device.

## 1. Introduction

The analysis of anterior segment structures is a main key for diagnosis and treatment of ocular disorders such us glaucoma or keratoconus as well as refractive surgery, corneal surgery, evaluation of visual quality, and fitting and development of contact lenses. Therefore, it is essential to use accurate instruments that provide a high repeatability of the different ocular parameters and in agreement with other devices [1–3].

The Galilei dual Scheimpflug analyzer (GDSA) (Ziemer Group, Port, Switzerland) is a noninvasive tool that combines the principles of two examination techniques, Placido rings and photography dual Scheimpflug camera, for the analysis of the anterior segment of the eye. The combination of these two technologies aims to get the advantages of both in a single scan and achieves more precise measurements [4–6].

This device allows a three-dimensional analysis of the anterior segment and obtains parameters of different structures, including corneal thickness, anterior and posterior corneal curvature, anterior and posterior corneal topography, corneal volume, anterior chamber depth, and horizontal and vertical distance from limbus to limbus [4–9]. Recent studies have shown a good repeatability of some of the parameters obtained with GDSA [7, 9–11]. There are other important parameters in clinical practice that are provided by GDSA but not studied in previous papers: anterior axial (AA) curvature average and posterior axial (PA) curvature average in three corneal sections, several instantaneous corneal parameters, refractive power (RP), and total power (TP) parameters.

Our study wants to provide a complete characterization of the anterior segment using GDSA and to investigate the repeatability of these indexes in normal Caucasian population using this device.

## 2. Material and Methods

Fifty-two eyes from 52 healthy volunteers were prospective and consecutively recruited from September 2011 to June 2012. The subjects were selected from the University of Zaragoza; all of them were students from the Optics and Optometry degree. The subjects underwent a full optometric examination in order to rule out any ocular pathology. One eye of each subject was randomly selected. The prospective study protocol was approved by the Clinical Research Ethics Committee of Aragón (CEICA) and written informed consent was obtained from all participants. The design of the study followed the tenets of the Declaration of Helsinki for biomedical research.

Exclusion criteria were previous ocular surgery, systemic diseases with ocular implications, the presence of keratitis or ocular inflammation, and current use of any medication that affects anterior segment parameters.

The Galilei system is able to obtain a three-dimensional reconstruction of the cornea and the anterior chamber. It is a noncontact method, and it has certain advantages over the ultrasound systems, as corneal anesthesia is not required, the procedure is comfortable for the patient, and that the risk of corneal abrasion or cross-infection between patients is minimum [12, 13].

During the scanning process, GDSA system (software version 5.2.1) acquires a series of 15/2 Scheimpflug 3D images and two Placido images with 90 degrees of separation that analyzes 122,000 points of the anterior segment of the eye. From the Scheimpflug imaging are detected the boundaries of anterior cornea, posterior cornea, lens, and iris. The main advantage of the dual Scheimpflug imaging is the corneal thickness calculation. The data obtained from both positions are averaged to compensate involuntary misalignment. The Scheimpflug principle is independent of inclined surfaces and therefore allows accurate measurement of the corneal thickness, although the distance between the slit and corneal apex is unknown.

The following values were evaluated in this study.

### 2.1. Anatomical Parameters

(1) Pachymetry: the arithmetic average of the pachymetry values is obtained in the central (0.0 mm to 2.0 mm), paracentral (2.0 mm to 3.5 mm), and peripheral section (3.5 mm to 5.0 mm) of the cornea. The Galilei system is able to find the thinnest point after detecting the area of 1.0 mm with average thinnest pachymetry.

(2) Corneal volume: the corneal volume is calculated over a diameter of 8 mm.

(3) Limbus: the nasal-temporal limbus and the superior-inferior limbus parameters are the maximum length in horizontal and vertical direction, respectively, of the best fit ellipse over the detected border of the limbus.

(4) Anterior chamber depth: it is the central distance between the corneal epithelium and the anterior surface of crystalline lens but Galilei really measures the aqueous depth, from the endothelium to the anterior surface of the lens. Thus, in this paper we use the term ACD like the measurement between endothelium and the crystalline lens.

(5) The anterior chamber volume is the volume of the space between the corneal endothelium and anterior lens and iris surface, respectively. Its volume is restricted to an 8 mm diameter due to Scheimpflug limitations.

(6) Iridocorneal angle measurement in each zone (temporal, nasal, superior, and inferior) is the average over a 30° section, respectively, to reduce the influence of outliers. Galilei calculates its anterior chamber angles by extrapolating edges that have been detected, because the Seimpflug systems cannot image through a nontransparent tissue as sclera.

### 2.2. Axial Parameters

(1) Anterior and posterior best fit sphere (BFS). The BFS is the spherical surface that best fits the shape of the examined cornea. Galilei provides the radius of this sphere.

(2) The elevation map provides the difference of elevation of each point of the cornea surface with respect to a BFS.

(3) Anterior and posterior SimK parameters: SimK steep (SimKs) and SimK flat (SimKf) are calculated from the pair of meridians 90° apart with the greatest difference in average power, from 0.5 to 2.0 mm radius on the anterior surface. SimKf is the flatter of the two meridians. SimKavg is the arithmetic average of the steep and the flat axis (SimKf, SimKs). For the anterior surface the index is calculated using the keratometric index (*n* = 1.3375) instead of the real refractive index of the cornea (*n* = 1.376). For the posterior surface Kf and Ks are exactly the same as SimKf and SimKs but taking into account the real refractive cornea index (*n* = 1.376).

(4) Anterior and posterior curvature average in, mid, and out: the central, paracentral, and peripheral average of corneal curvature are the arithmetic average of the curvature values in the corresponding section of the cornea. The central section is already defined previously.

### 2.3. Instantaneous Parameters

Instantaneous maps show detailed patterns revealing a more exact location of a corneal defect when compared to an axial map. Axial corneal topography measures all curvatures relative to the corneal topography axis and provides a global description of shape. The instantaneous radius of curvature map has no reference axis and only determines the curvature local radius at each point [14].

Anterior and posterior eccentricity (*e* ^2^): actually the eccentricity is reported as its square and it is corresponded with corneal shape factor (*E*). These terms are parameters to describe the shape of a conic section. These terms are mathematically related by the following equation: *E* = *e* ^2^ = −*Q*. Galilei calculates the eccentricity *e* ^2^ of the surface within a central diameter of 8 mm averaged over all meridians.

### 2.4. Refractive Power

RP only uses data from the anterior surface whereas TCP uses data from the total cornea (anterior and posterior) over radius from 0.5 mm to 2.0 mm radius. Flat, steep, and average indexes are calculated from the pair of meridians 90° apart with the greatest difference in RP or TCP. Average in, mid, and out have been previously defined [15].

### 2.5. Statistical Analyses

Statistical analyses were carried out with the Statistical Package for the Social Sciences (SPSS 20.0, SPSS Inc., Chicago, IL, USA). The Shapiro-Wilk test was used to assess the normal distribution of all quantitative parameters. Results were defined by the mean and standard deviation of each parameter and the mean of three measurements. Results and their differences were analyzed using the analysis of variance (ANOVA) test for the comparison of means and values of *P* < 0.05 which were considered to be indicative of statistically significant differences. To assess the repeatability of repeated measurements ANOVA was also performed to determinate the within subject standard deviation (*S*_*w*_). The *S*_*w*_ is the repeatability of the measurements and the following methods were used [16–18].

## 3. Results

### 3.1. Demographic Parameters

Twenty-six right eyes and 26 left eyes of 52 subjects were analyzed; 14 subjects (27%) were men and 38 (73%) were women. The mean age was 23 years, range 22 to 28 years. Tables 1 to 4 show the mean value of each parameter and SD for the three measurements. Repeated measures of ANOVA did not detect significant differences between the three consecutive measurements for any of the studied parameters.

### 3.2. Intrasubject Repeatability for Anatomical Parameters of the Anterior Segment

Table 1 shows the outcomes of the intrasubject repeatability for the measurements of anatomical parameters of the anterior chamber. Regarding ICC, all parameters showed almost perfect reliability (ICC > 0.81) except thinnest CCT (0.78) and corneal thickness average out (0.79). All parameters had low COV (<10%) except anterior chamber volume and superior iridocorneal angle, both of them were with high repeatability limits (52.30 mm^3^ and 10.46°), respectively.

### 3.3. Intrasubject Repeatability for Curvature

Table 2 shows mean axial measurements relative to the anterior and posterior corneal surfaces. COV, ICC, and test-retest variability results demonstrated high repeatability with COV lower than 5% and almost perfect reliability (ICC > 0.81) for all parameters, the only exception was posterior axial curvature average out with ICC = 0.60.

Table 3 shows mean local radius of curvature at each corneal point (instantaneous parameters) and eccentricity. COV, ICC, and test-retest variability results demonstrated high repeatability with COV lower than 5% and almost perfect reliability (ICC > 0.81) for most of the parameters. Exceptions were AI Curvature *e* ^2^ (ICC = 0.81; COV = 66.67%) and PI Curvature *e* ^2^ (ICC = 0.88; COV = 127.78%); their extremely high COVs are due to the proximity to zero of the mean value of the parameters and its sensitivity to changes. PI curvature average mid and PI curvature average out had substantial reliability ICC (ICC > 0.61) with worse COV (5.58% and 8.86%, resp.). AI curvature average out had the worst ICC = 0.55 and COV = 6.62%, although it can be considered moderate reliability.

Concerning refractive power (anterior corneal surface) and total corneal power ray traced (anterior and posterior corneal surfaces), ICC reached values close to 1 and COV < 3.5% (Table 4).

## 4. Discussion

Noncontact ocular anterior segment topography has been developed because of its advantages versus contact techniques: it does not require the use of topical anesthesia and a small risk for corneal infection is known. An ophthalmic device must provide correlation among repeated measurements (ICC) and good repeatability that indicates the ability to obtain the same values repeated measurements under similar conditions. Repeatability should be described in terms of repeatability (*S*_*w*_ or COV) and repeatability limits (*R*), reported as 2.77*S*_*w*_ which gives the likely limits with which 95% or measurements should be within.

In this study, we evaluated these statistical indicators of anterior segment measurements with a GDSA that combines Scheimpflug photography and Placido-disc. Few parameters of the Galilei and a complete study of repeatability have been evaluated in previous studies; we have evaluated most of the parameters that this topography provides.

This study showed that dual Scheimpflug system has high correlation among repeated measurements for corneal thickness average in a 3.5 mm section, with ICC very close to 1, but we found worse results in the paracentral zone which comprised between 3.5 mm and 5.0 mm. In the central zone, our results are better than previous publications [7–11], similar in paracentral zone [11], and worse in the peripheral zone [11]. Corneal thickness average varied almost 130 *μ*m from the central to the outer sector but the COV remained constant and under the 5% for the three zones. It can be considered an indication of high repeatability but with better ICC in the inner than in the outer zone. Although the dual channel rotating Scheimplug cameras improve the measurements avoiding decentrations or different thicknesses, its ability for measuring peripheral zones has some limitations.

The ICC of thinnest CCT and corneal diameters were slightly lower than the values obtained by Savini et al. [10] or Aramberri et al. [9]. Specifically, our thinnest CCT result is due to the high value of the *S*_*w*_ caused by the variation of the individual standard deviations from 6.77% to 10.63% of the mean value. Our COV was higher than in the other anatomical parameters but remained below 10% so it is still indicating a good repeatability. However, the repeatability limit *R* shows poor precision of the device to measure pachymetry.

Excellent correlation among repeated measurements were found in anterior chamber depth and iridocorneal angles as it had been already reported [5, 9, 10, 19]. Anterior chamber volume and superior iridocorneal angle deserve special attention because their COV were higher than 10%. In both parameters this COV and the limits of agreement method (*R*) reveal poor precision unlikely to have good agreement among repeated measurements.

In our study, GDSA demonstrated an excellent correlation and repeatability in measuring axial, refractive, and total corneal powers but some instantaneous parameters tended to worse values. Mean axial corneal power decreased from center to periphery; the within-subject SD was less than 1.4 D for all anterior axial regions and 0.3 D for posterior axial regions. The same decreasing tendency showed the anterior and posterior instantaneous corneal powers. but the *S*_*w*_ was higher in these cases, 2.6 D and 0.5 D maximum, respectively; a reason for this finding could be the difficulty to determinate the local radius of curvature at each point in the peripheral zone diameter. Despite this, the worst ICC found was 0.55 for the outer anterior curvature average. Central and paracentral powers had similar repeatability than their equivalent axial parameters.

The mean refractive power and the total corneal power increased from center to periphery by a mean of 1.37 D (*S*_*w*_ < 1.5 D) and 0.81 D (*S*_*w*_ < 1.4 D), respectively. The mean TCP results and their ICC values were similar to the readings reported by Wang et al. [11] although within-subject SD, repeatability and COV of this previous paper were higher than our results. Menassa et al. [7] and Shirayama et al. [20] also stated that both the steep Ks and flat Kf meridians had remarkably low intraobserver variation with ICCs consistent with our findings.

Anterior (axial and instantaneous), RP and TC had excellent COV values of SimK parameters lower than 3.5%, while posterior (axial and instantaneous) varied from 4.10% to 8.86% demonstrating worse repeatability of the posterior corneal surface and peripheral points measurements. Eccentricity value was also an instantaneous parameter with high variability. The found results can indicate that variation in measurements was mainly due to true subject-to-subject variation rather than observer error.

If we compare our results with those obtained with other devices with dual Scheimpflug camera for different parameters, Sirius (Costruzione Strumenti Oftalmici) and Pentacam (Oculus Optikgeräte GmbH), good repeatability for these instruments were found [21–23].

In conclusion, we found the GDSA in healthy young persons had an almost perfect correlation in measuring anatomic, axial, instantaneous, and refractive parameters with greater variability for peripheral terms. However, for anatomical parameters like pachymetry, anterior chamber, or iridocorneal angle and eccentricity its repeatability and repeatability limits were poor. These results suggested that more caution must be taken during the measure of certain corneal parameters because it cannot be repeatable. In healthy young persons, the other evaluated parameters had very good repeatability and their limits of agreement showed excellent clinical results for this device.

## Figures and Tables

**Table 1:** Mean pachymetric, white-to-white and anterior chamber values and standard deviations, ANOVA test, repeatability, intraclass coefficients, and coefficients of variation in healthy eyes.

Anatomical parameters	Mean_1_ ± SD	Mean_2_ ± SD	Mean_3_ ± SD	*P* ANOVA	Mean ± Sw	*R*	ICC (lower 95% CI)	COV (%)
Thinnest CCT (*μ*m)	548.96 ± 51.66	552.07 ± 37.37	547.85 ± 58.22	0.460	551.36 ± 45.51	126.06	0.78 (0.66)	8.25
Corneal thickness average in (*μ*m)	568.53 ± 26.80	569.29 ± 26.68	568.88 ± 26.91	0.066	568.91 ± 26.76	74.13	1.00 (0.99)	4.71
Corneal thickness average mid (*μ*m)	616.02 ± 26.35	616.68 ± 26.97	616.19 ± 26.93	0.779	616.29 ± 26.44	73.23	0.99 (0.98)	4.29
Corneal thickness average out (*μ*m)	700.12 ± 36.59	696.25 ± 43.09	698.95 ± 32.43	0.728	698.44 ± 31.41	87.00	0.79 (0.66)	4.50
Corneal volume (mm^3^)	34.32 ± 1.47	34.33 ± 1.51	34.32 ± 1.48	0.995	34.32 ± 1.46	4.03	0.98 (0.97)	4.24
Limbo nasal-temporal (mm)	12.43 ± 0.52	12.35 ± 0.50	12.40 ± 0.43	0.387	12.38 ± 0.47	1.29	0.94 (0.88)	3.78
Limbo superior-inferior (mm)	12.10 ± 0.52	12.01 ± 0.52	12.00 ± 0.57	0.283	12.04 ± 0.50	1.38	0.84 (0.71)	4.14
ACD (mm)	3.30 ± 0.27	3.28 ± 0.27	3.29 ± 0.27	0.192	3.29 ± 0.27	0.74	1.00 (0.99)	8.12
Anterior chamber volume (mm^3^)	113.83 ± 20.95	118.84 ± 20.58	117.68 ± 27.88	0.054	117.67 ± 18.88	52.30	0.93 (0.88)	16.05
Temporal Iridocorneal angle (degrees)	37.62 ± 2.87	37.50 ± 2.88	37.67 ± 2.97	0.349	37.60 ± 2.85	7.89	0.99 (0.98)	7.58
Superior Iridocorneal angle (degrees)	37.07 ± 4.01	36.58 ± 3.83	36.51 ± 4.12	0.127	36.72 ± 3.78	10.46	0.95 (0.92)	10.29
Nasal Iridocorneal angle (degrees)	37.23 ± 3.18	37.34 ± 3.17	37.22 ± 3.19	0.300	37.29 ± 3.13	8.66	0.99 (0.98)	8.38
Inferior Iridocorneal angle (degrees)	38.80 ± 3.70	38.41 ± 3.60	38.45 ± 3.74	0.104	38.55 ± 3.58	9.93	0.97 (0.96)	9.30

CCT: central corneal thickness; ACD: anterior chamber deep from the corneal endothelium; SD: standard deviation; Sw: within-subject SD; *P*: ANOVA-test (values of *P* < 0.05 were considered to be indicative of significant differences); *R*: repeatability limits; ICC: intraclass coefficient; COV: coefficient of variation.

**Table 2:** Mean axial corneal parameters and standard deviations, ANOVA test, repeatability, intraclass coefficients, and coefficients of variation in healthy eyes.

Axial parameters	Mean_1_ ± SD	Mean_2_ ± SD	Mean_3_ ± SD	*P* ANOVA	Mean ± Sw	*R*	ICC (lower 95% CI)	COV (%)
Anterior best fit sphere (mm)	7.78 ± 0.25	7.78 ± 0.25	7.78 ± 0.24	0.286	7.78 ± 0.25	0.68	1.00 (0.95)	3.14
AA SimK steepest (D)	44.22 ± 1.43	44.23 ± 1.45	44.13 ± 1.53	0.448	44.19 ± 1.43	3.95	0.97 (0.95)	3.23
AA SimK flattest (D)	43.43 ± 1.42	43.40 ± 1.35	43.34 ± 1.58	0.666	43.39 ± 1.40	3.87	0.96 (0.94)	3.22
AA SimK average (D)	43.82 ± 1.40	43.82 ± 1.38	43.74 ± 1.53	0.543	43.79 ± 1.39	3.86	0.97 (0.95)	3.18
AA curvature average in (D)	43.84 ± 1.40	43.84 ± 1.36	43.75 ± 1.55	0.518	43.81 ± 1.39	3.84	0.96 (0.94)	3.17
AA Curvature average mid (D)	43.38 ± 1.32	43.38 ± 1.39	43.35 ± 1.32	0.408	43.37 ± 1.34	3.71	1.00 (0.99)	3.09
AA curvature average out (D)	42.57 ± 1.21	42.56 ± 1.22	42.59 ± 1.28	0.881	42.57 ± 1.20	3.32	0.97 (0.96)	2.82
Posterior best fit sphere (mm)	6.39 ± 0.29	6.42 ± 0.25	6.41 ± 0.25	0.172	6.41 ± 0.26	0.70	0.96 (0.94)	3.97
PA SimK steepest (D)	−6.51 ± 0.29	−6.54 ± 0.37	−6.51 ± 0.31	0.356	−6.52 ± 0.31	0.85	0.93 (0.89)	4.68
PA SimK flattest (D)	−6.17 ± 0.25	−6.18 ± 0.26	−6.17 ± 0.28	0.589	−6.17 ± 0.26	0.71	0.98 (0.96)	4.14
PA SimK average (D)	−6.34 ± 0.27	−6.36 ± 0.30	−6.34 ± 0.29	0.356	−6.35 ± 0.28	0.76	0.96 (0.94)	4.32
PA curvature average in (D)	−6.34 ± 0.27	−6.37 ± 0.30	−6.35 ± 0.29	0.438	−6.35 ± 0.28	0.77	0.95 (0.93)	4.36
PA curvature average mid (D)	−6.28 ± 0.29	−6.23 ± 0.25	−6.26 ± 0.24	0.133	−6.25 ± 0.24	0.67	0.92 (0.87)	3.86
PA curvature average out (D)	−6.11 ± 0.28	−6.01 ± 0.46	−6.08 ± 0.22	0.152	−6.07 ± 0.25	0.69	0.60 (0.38)	4.10

AA: anterior axial; PA: posterior axial; SD: standard deviation; Sw: within-subject SD; *P*: ANOVA-test (values of *P* < 0.05 were considered to be indicative of significant differences); *R*: repeatability limits; ICC: intraclass coefficient; COV: coefficient of variation.

**Table 3:** Mean instantaneous corneal parameters and standard deviations, ANOVA test, repeatability, intraclass coefficients, and coefficients of variation in healthy eyes.

Instantaneous Parameters	Mean_1_ ± SD	Mean_2_ ± SD	Mean_3_ ± SD	*P* ANOVA	Mean ± Sw	*R*	ICC (lower 95% CI)	COV (%)
AI SimK Steepest (D)	44.12 ± 1.44	44.16 ± 1.81	44.03 ± 1.48	0.426	44.10 ± 1.53	4.24	0.96 (0.95)	3.47
AI SimK flattest (D)	43.27 ± 1.48	43.23 ± 1.49	43.17 ± 1.42	0.097	43.22 ± 1.45	4.02	0.99 (0.98)	3.36
AI SimK average (D)	43.69 ± 1.43	43.69 ± 1.62	43.60 ± 1.42	0.277	43.66 ± 1.47	3.86	0.98 (0.97)	3.36
AI curvature average in (D)	43.73 ± 1.38	43.73 ± 1.44	43.67 ± 1.41	0.337	43.71 ± 1.39	3.46	0.99 (0.98)	3.19
AI curvature average mid (D)	41.70 ± 1.21	41.70 ± 1.29	41.81 ± 1.53	0.565	41.74 ± 1.25	7.06	0.92 (0.87)	2.99
AI curvature average out (D)	38.17 ± 2.43	38.14 ± 2.18	38.45 ± 3.02	0.458	38.50 ± 2.55	7.07	0.55 (0.29)	6.62
AI curvature *e* ^2^	0.23 ± 0.16	0.24 ± 0.16	0.25 ± 0.17	0.571	0.24 ± 0.16	0.45	0.81 (0.70)	66.67
PI curvature average in (D)	−6.34 ± 0.28	−6.33 ± 0.25	−6.33 ± 0.26	0.690	−6.33 ± 0.26	0.72	0.98 (0.97)	4.10
PI curvature average mid (D)	−5.95 ± 0.37	−5.32 ± 0.30	−5.91 ± 0.29	0.571	−5.92 ± 0.33	0.92	0.71 (0.53)	5.58
PI curvature average out (D)	−5.18 ± 0.35	−5.22 ± 0.47	−5.14 ± 0.55	0.627	−5.19 ± 0.46	1.28	0.66 (045)	8.86
PI curvature *e* ^2^	0.19 ± 0.23	0.18 ± 0.23	0.22 ± 0.25	0.638	0.18 ± 0.23	0.64	0.88 (0.81)	127.78

AI: anterior instantaneous; PI: posterior instantaneous; SD: standard deviation; Sw: within-subject SD; *P*: ANOVA-test (values of *P* < 0.05 were considered to be indicative of significant differences); *R*: repeatability limits; ICC: intraclass coefficient; COV: coefficient of variation.

**Table 4:** Mean refractive power, total corneal power (ray traced) and standard deviations, ANOVA test, repeatability, intraclass coefficients, and coefficients of variation in healthy eyes.

Refractive power and Total corneal power	Mean_1_ ± SD	Mean_2_ ± SD	Mean_3_ ± SD	*P* ANOVA	Mean ± Sw	*R*	ICC (lower 95% CI)	COV (%)
RP SimK steepest (D)	44.57 ± 1.47	44.58 ± 1.49	44.47 ± 1.56	0.446	44.54 ± 1.46	4.05	0.97 (0.95)	3.28
RP SimK flattest (D)	43.76 ± 1.45	43.73 ± 1.39	43.67 ± 1.61	0.665	43.72 ± 1.43	3.97	0.96 (0.94)	3.28
RP SimK average (D)	44.16 ± 1.44	44.15 ± 1.41	44.07 ± 1.57	0.541	44.13 ± 1.43	3.95	0.97 (0.95)	3.23
RP average in (D)	44.10 ± 1.42	44.09 ± 1.39	44.00 ± 1.58	0.515	44.06 ± 1.41	3.91	0.96 (0.94)	3.21
RP average mid (D)	44.96 ± 1.47	44.95 ± 1.55	44.92 ± 1.48	0.436	44.94 ± 1.49	4.13	1.00 (0.99)	3.32
RP average out (D)	45.42 ± 1.47	45.41 ± 1.48	45.45 ± 1.55	0.864	45.43 ± 1.46	4.05	0.97 (0.96)	3.22
TCP (RT) SimK steepest (D)	42.37 ± 1.34	42.39 ± 1.38	42.27 ± 1.44	0.327	42.34 ± 1.34	3.72	0.97 (0.95)	3.17
TCP (RT) SimK flattest (D)	41.70 ± 1.33	41.63 ± 1.27	41.60 ± 1.48	0.606	41.64 ± 1.30	3.59	0.95 (0.92)	3.11
TCP (RT) SimK average (D)	42.03 ± 1.32	42.01 ± 1.29	41.93 ± 1.44	0.507	41.99 ± 1.30	3.61	0.96 (0.94)	3.10
TCP (RT) average in (D)	41.99 ± 1.31	41.96 ± 1.27	41.89 ± 1.47	0.490	41.95 ± 1.29	3.58	0.96 (0.93)	3.09
TCP (RT) average mid (D)	42.54 ± 1.28	42.58 ± 1.47	42.53 ± 1.34	0.528	42.55 ± 1.35	3.74	0.99 (0.98)	3.17
TCP (RT) average out (D)	42.71 ± 1.28	42.78 ± 1.34	42.80 ± 1.41	0.646	42.76 ± 1.28	3.54	0.95 (0.92)	2.99

RP: refractive power; TCP (RT): total corneal power (ray traced); SD: standard deviation; Sw: within-subject SD; *P*: ANOVA-test (values of *P* < 0.05 were considered to be indicative of significant differences); *R*: repeatability limits; ICC: intraclass coefficient; COV: coefficient of variation.
